# Employment of color Doppler echocardiographic video clips in a cardiac auscultation class with a cardiology patient simulator: discrepancy between students’ satisfaction and learning

**DOI:** 10.1186/s12909-021-03033-8

**Published:** 2021-12-06

**Authors:** Yutaka Kagaya, Masao Tabata, Yutaro Arata, Junichi Kameoka, Seiichi Ishii

**Affiliations:** 1 Faculty of Medical Science and Welfare, Tohoku Bunka Gakuen University, 6-45-1, Kunimi, Aoba-ku, Sendai, 981-8551 Japan; 2 Patient Safety Management Office, Tohoku University Hospital, Sendai, Japan; 3 Graduate Medical Education Center, Tohoku University Hospital, Sendai, Japan; 4 Division of Hematology and Rheumatology, Faculty of Medicine, Tohoku Medical and Pharmaceutical University, Sendai, Japan; 5 Office of Medical Education, Tohoku University Graduate School of Medicine, Sendai, Japan

**Keywords:** Cardiac auscultation, Cardiology patient simulator, Color-Doppler echocardiography, Heart murmurs, Heart sounds

## Abstract

**Background:**

We have provided fourth-year medical students with a three-hour cardiac auscultation class using a cardiology patient simulator since 2010. The test results of 2010-2012 revealed that as compared with aortic stenosis murmur, students correctly identified murmurs of other valvular diseases less often. We investigated whether employment of color Doppler echocardiographic video clips would improve proficiency in identifying murmurs of aortic regurgitation and mitral regurgitation, and whether students’ favorable responses to a questionnaire were associated with improved proficiency.

**Methods:**

A total of 250 fourth-year medical students were divided into groups of 7-9 students in 2014 and 2015. Each group attended a three-hour cardiac auscultation class comprising a mini-lecture, facilitated training, two different auscultation tests (the second test being closer to clinical setting than the first) and a questionnaire. We provided each student with color Doppler echocardiographic videos of aortic regurgitation and mitral regurgitation using a tablet computer, which they freely referred to before and after listening to corresponding murmurs. The test results were compared with those in 2010-2012. The students had already completed the course of cardiovascular medicine, comprising lectures including those of physical examination, echocardiography, and valvular heart diseases, before participating in this auscultation training class.

**Results:**

Most students indicated that the videos were useful or somewhat useful regarding aortic regurgitation (86.3%) and mitral regurgitation (85.7%). The accuracy rates were 78.4% (81.2% in 2010-2012) in aortic regurgitation and 76.0% (77.8%) in mitral regurgitation in the first test, and 83.3% (71.4%) in aortic regurgitation and 77.1% (77.6%) in mitral regurgitation in the second test, showing no significant differences as compared to 2010-2012. Overall accuracy rate of all heart sounds and murmurs in the first test and that of second/third/fourth sounds in the first and second tests were significantly lower in 2014-2015 than in 2010-2012.

**Conclusions:**

Referring to color Doppler echocardiographic video clips in the way employed in the present study, which most students regarded as useful, did not improve their proficiency in identifying the two important regurgitant murmurs, revealing a discrepancy between students’ satisfaction and learning. Video clips synchronized with their corresponding murmurs may contribute toward improving students’ proficiency.

## Background

Although there is a possibility that stethoscopes will be replaced by a handheld ultrasound device in the distant future [1], cardiac auscultation is an essential physical-examination skill to noninvasively diagnose patients with a variety of heart diseases without any expensive equipment [2]. Medical students in Japan must pass the objective structured clinical examination (OSCE) organized by the Common Achievement Tests Organization, a public interest incorporated association in Japan before starting their clinical clerkship program in their fourth or fifth year of six-year undergraduate medical education [3, 4]. The Common Achievement Tests Organization has determined essential proficiencies that medical students must acquire before participating in the clinical clerkship. With regard to cardiac auscultation, students have to be able to identify split second sound (S2) and third (S3) and fourth (S4) sounds as well as systolic and diastolic heart murmurs. Although cardiology patient simulators are widely used to provide medical students with an opportunity to learn these heart sounds and murmurs, it is still unclear whether expensive simulators are really beneficial [5–14]. Therefore, we need to develop an auscultation training program that substantially improves proficiency of medical students in accurately identifying heart sounds and murmurs.

We have provided fourth-year medical students at Tohoku University School of Medicine with a 3-h cardiac auscultation class using a cardiology patient simulator since 2010. From 2010 to 2012, a total of 324 students, divided into groups of 6-8 students, participated in the class [15]. Using two different types of auscultation test in the class, we demonstrated that the medical students were less likely to correctly identify S2/S3/S4 as compared with heart murmurs in a situation close to clinical setting. We also found that as compared with the murmur of aortic stenosis (AS), students correctly identified the murmurs of other valvular heart diseases, namely aortic regurgitation (AR), mitral regurgitation (MR), and mitral stenosis (MS), less often, although the accuracy rates of heart murmurs were higher than those of S2/S3/S4.

In 2013, we tried to test the hypothesis that providing echocardiographic color Doppler video clips of AR and MR using a single 42-in. display and encouraging the students to refer to them before and after listening to AR and MR murmurs may improve their proficiency in identifying them. However, we found it difficult to have them sufficiently watch the display in the class. Therefore, in 2014 and 2015, we provided each student with echocardiographic color Doppler video clips of AR and MR using a tablet computer, which they freely referred to before and after listening to corresponding murmurs of a cardiology patient simulator. We then investigated whether referring to the video clips on the tablet before and after listening to the murmurs would improve proficiency in identifying murmurs of AR and MR of a cardiology patient simulator by comparing the auscultation test results with those in 2010-2012. We also asked the students to answer a questionnaire at the end of the class regarding the usefulness of referring to color Doppler echocardiographic video clips, and investigated whether favorable responses, if any, were associated with an improvement in their proficiency in identifying the two important regurgitant murmurs.

## Methods

### Participants and cardiac auscultation training class

This study was conducted in a three-hour cardiac auscultation training class [15] using four cardiology patient simulators (“K”®, Kyoto-Kagaku, Kyoto, Japan) [16]. The students had already completed the course of cardiovascular medicine, comprising lectures including those of physical examination, echocardiography and valvular heart diseases, before participating in this auscultation training class. A total of 250 fourth-year medical students (121 students in 2014 and 129 students in 2015) at Tohoku University School of Medicine were divided into groups of 7-9 students. Each group visited the Tohoku University Clinical Skills Laboratory in turn on Wednesday afternoons from September to December, and the students joined a three-hour cardiac auscultation class using a cardiology patient simulator. This cardiac auscultation class was part of a rotational clinical-skills training course to prepare students for clinical clerkship, which started at the beginning of their fifth year of six-year undergraduate medical education. We obtained approval from the Ethical Committee of Tohoku University Graduate School of Medicine. Details of the rotational clinical-skills training course including the cardiac auscultation training class are described in our previously published article [15].

Two teachers (Y.K. and M.T.) were in charge of the three-hour cardiac auscultation class. The class consisted of a mini-lecture, facilitated training, self-training, two different auscultation tests and feedback, which were exactly the same as those provided in 2010-2012 [15] except that color Doppler echocardiographic video clips of AR and MR were provided using a tablet computer as described in detail below. The heart sounds and murmurs which we focused on in the class were non-split S2, respiratory split S2, abnormally wide split S2, S3, S4 and four different heart murmurs, AS, AR, MR and MS, which were classified into three categories as shown in Table 1. We chose only these four valvular heart diseases because we believed that they were associated with representative systolic or diastolic heart murmurs and time assigned to our cardiac auscultation class was limited. Therefore, we were not able to employ other important sounds such as innocent murmurs or systolic clicks.

**Table 1:** Heart sounds and heart murmurs studied in the class in both 2010-2012 and 2014-2015

Category A	Category B	Category C
Non-split S2	S3	Aortic stenosis
Respiratory split S2	S4	Aortic regurgitation
Abnormally wide split S2	S3 + S4	Mitral regurgitation
		Mitral stenosis

S2, second sound; S3, third sound; S4, fourth sound

### Echocardiographic color Doppler video clips

We provided each student with echocardiographic color Doppler video clips of AR and MR using a tablet computer (iPad mini®, Apple Inc., Cupertino, U.S.A), which they freely referred to before and after listening to corresponding murmurs. These video clips of AR and MR were recorded from typical patients of AR and MR, respectively, and had no relationship with the AR and MR murmurs of the cardiology patient simulators. We encouraged the students to refer to the video clips before and after listening to corresponding heart murmurs while imagining the sound of the murmur.

### Two different cardiac auscultation tests

**Fig. 1:**
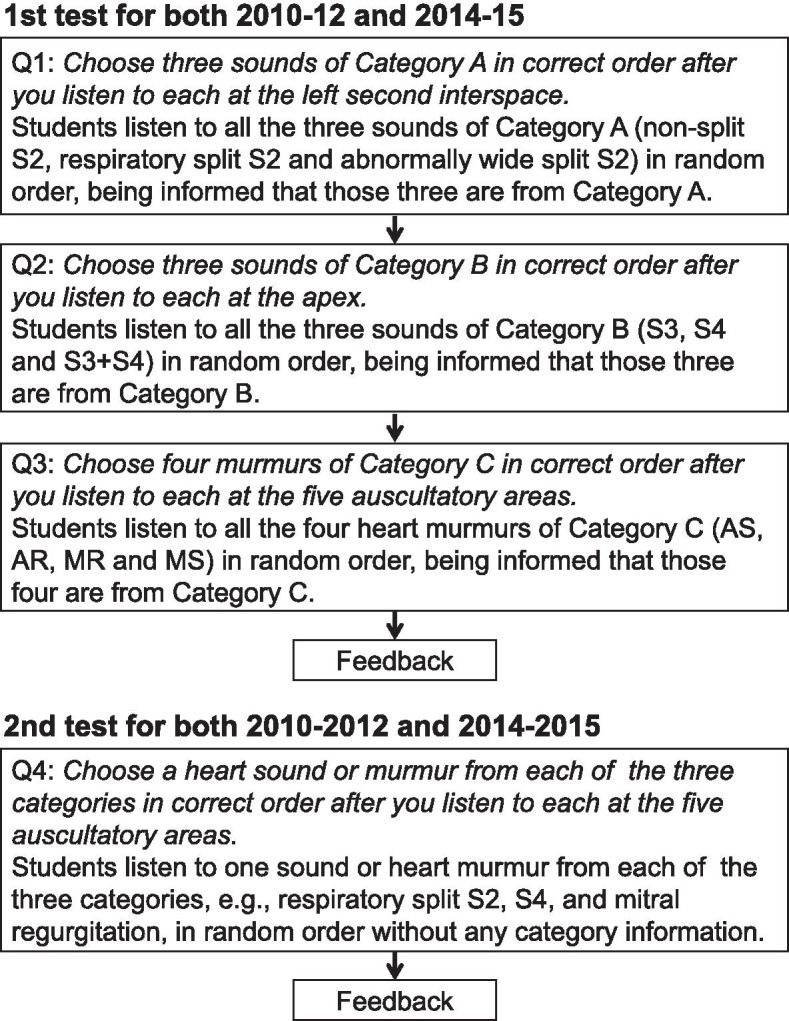
Protocols of the two different auscultation tests in both 2010-2012 and 2014-2015. S2, second sound; S3, third sound; S4, fourth sound; AS, aortic stenosis; AR, aortic regurgitation; MR, mitral regurgitation; MS, mitral stenosis. The five auscultatory areas represent the right second interspace, left second interspace, left third interspace, left fourth or fifth interspace and apex

Two different cardiac auscultation tests were provided for the students in exactly the same way as in 2010-2012 as described in our previous study [15] (Fig. 1). The students were informed that their performance in the two tests would have no bearing on their grades in the rotational clinical-skills training course. All the students consented to participate in the study. In the first test, the students were informed that the sounds they were going to listen to belonged to Category A. The students then listened to the three heart sounds in Category A in random order and wrote their answer. Next, the students listened to three heart sounds in Category B in random order and wrote their answers again after being informed that the sounds were from Category B. Finally, they answered in exactly the same way with regard to Category C. Since we gave feedback to the students soon after the first test, they were able to know which heart sounds or murmurs they correctly identified and which ones they did not before taking the second test.

The second test was closer to a situation in a real clinical setting than the first test, as in the second test, we had students listen to only one heart sound or murmur from each of the three categories in random order without informing the students of the categories before they listened to them. The students then tried to identify the two heart sounds and the murmur. We randomly selected these two heart sounds and one heart murmur from each of the three categories. The teachers gave feedback to the students immediately after the second test.

### Likert scale responses of the students

Students’ 5-point Likert scale responses regarding the usefulness of color Doppler echocardiographic video clips of AR and MR in the cardiac auscultation class were obtained at the end of the class using a questionnaire.

### Statistical analysis

Comparisons of the accuracy of heart sounds and murmurs between 2010 and 2012 and 2014-2015 were performed by Fisher’s exact test using JMP Pro 15.2.0 software (SAS Institute Inc., Cary, NC, U.S.A.). We considered a *p*-value of < 0.05 as statistically significant.

## Results

Accuracy rates of heart sounds and heart murmurs in the first test and second test obtained in 2010-2012 and 2014-2015 are summarized in Table 2. Overall accuracy rate of all heart sounds and heart murmurs in the first test was significantly lower in 2014-2015 than in 2010-2012. That in the second test tended to be lower in 2014-2015 than in 2010-2012, but the difference was not statistically significant. The accuracy rate of S2/S3/S4 was significantly lower in 2014-2015 than in 2010-2012 in both the first test and the second test. The accuracy rate of heart murmurs was not significantly different between 2010-2012 and 2014-2015 either in the first test or in the second test. The accuracy rate of respiratory split S2 in the first test was significantly lower and that of S3 in the second test tended to be lower in 2014-2015 than in 2010-2012. The accuracy rates of all the other heart sounds and heart murmurs were not significantly different between 2010-2012 and 2014-2015 either in the first test or in the second test including AR and MR despite the employment of color Doppler echocardiographic video clips of AR and MR in 2014-2015. Regarding AR in 2014-2015, wrong answers were AS (10.0% of total answers in the first test, 9.3% in the second test), MR (3.6, 3.7%), and MS (8.0, 3.7%). As to MR in 2014-2015, wrong answers were AS (4.4% of total answers in the first test, 5.7% in the second test), AR (4.8, 0%), and MS (14.8, 17.1%). Wrong answers for AR and MR in 2010-2012 were similar to those in 2014-2015 except that students were significantly less likely to mistake AS for AR in the first test in 2010-2012 than in 2014-2015 (5.2% vs 10.0%, *P* = 0.036).

**Table 2:** Accuracy rates in the first test and second test in 2010-2012 and 2014-2015

	First test			Second test		
	Accuracy rate (%), (n)		*P*	Accuracy rate (%), (n)		*P*
Years	2010-2012	2014-2015		2010-2012	2014-2015	
All	80.4 (3240)	77.4 (2500)	0.0059	62.0 (972)	57.5 (750)	0.059
S2/S3/S4	79.8 (1944)	76.7 (1500)	0.030	54.3 (648)	47.8 (500)	0.032
Heart murmurs	81.3 (1296)	78.5 (1000)	0.10	77.5 (324)	76.8 (250)	0.92
Non-split S2	87.4 (324)	85.6 (250)	0.54	50.9 (106)	46.5 (86)	0.56
Respiratory split S2	86.1 (324)	79.6 (250)	0.043	58.8 (114)	54.4 (79)	0.56
Abnormally split S2	92.6 (324)	92.0 (250)	0.87	59.6 (104)	50.6 (85)	0.24
S3	73.5 (324)	69.6 (250)	0.35	49.1 (114)	35.3 (85)	0.061
S4	72.5 (324)	69.6 (250)	0.46	65.4 (104)	66.3 (86)	1.0
S3 + S4	67.0 (324)	64.0 (250)	0.48	42.5 (106)	32.9 (79)	0.22
AS	88.9 (324)	84.0 (250)	0.11	94.8 (77)	88.7 (62)	0.22
AR	81.2 (324)	78.4 (250)	0.46	71.4 (84)	83.3 (54)	0.15
MR	77.8 (324)	76.0 (250)	0.62	77.6 (85)	77.1 (70)	1.0
MS	77.5 (324)	75.6 (250)	0.62	66.7 (78)	59.4 (64)	0.39

All, all heart sounds and heart murmurs; S2, second sound; S3, third sound; S4, fourth sound; AS, aortic stenosis; AR, aortic regurgitation; MR, mitral regurgitation; MS, mitral stenosis. Since 324 students in 2010-2012 and 250 students in 2014-2015 listened to three heart sounds of Category A, three heart sounds of Category B and four heart murmurs of Category C in the first test and one heart sound of Category A, one heart sound of Category B and one murmur of Category C in the second test, the number of heart sounds or murmurs that the students listened to was as shown in the parenthesis of each cell. The number was approximately one-third for each heart sound in Categories A and B and one-fourth for each murmur in Category C in the second test as compared with the first test because of the differences in the number of each heart sound or murmur randomly assigned to the students between the two tests. The data of 2010-2012 have been reported in our previously published article [15]

**Fig. 2:**
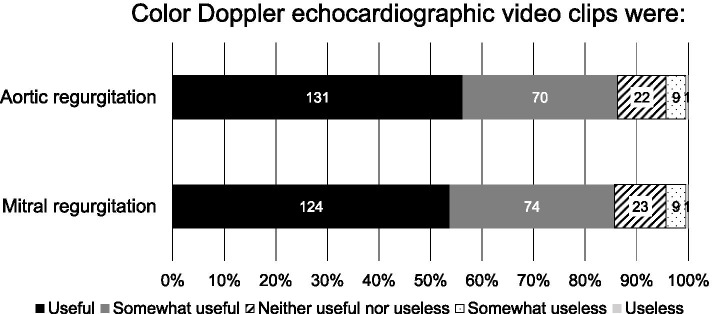
Questionnaire responses of the students in 2014-2015. The numbers in the columns represent the numbers of students who gave corresponding choices

Out of 250 students who participated in the cardiac auscultation class using a cardiology patient simulator in 2014-2015, 233 students (93.2%) and 231 students (92.4%) responded to the 5-point Likert scale questionnaire with regard to AR and MR, respectively. As shown in Fig. 2, 86.2% of the students answered that referring to color Doppler echocardiographic video clips in the cardiac auscultation class was useful or somewhat useful with regard to AR and 85.7% of the students answered that it was useful or somewhat useful with regard to MR.

## Discussion

We demonstrated that referring to color Doppler echocardiographic video clips of AR and MR in the way employed in the present study, which most students regarded as useful, did not improve their proficiency in correctly identifying the two important regurgitant murmurs, revealing a discrepancy between students’ satisfaction and learning.

### No beneficial effects of referring to color Doppler echocardiographic video clips in the way employed in the present study on proficiency in identifying AR and MR murmurs

We provided our medical students with color Doppler echocardiographic video clips of AR and MR using a tablet computer so that they were able to freely refer to them before and after they listened to corresponding murmurs of a cardiology patient simulator. We frequently encouraged the students to refer to the video clips while imagining the sound of the corresponding heart murmurs. The reason that we decided to employ these video clips was that we had found in our previous study that medical students were less likely to accurately identify AR, MR, and MS murmurs than AS murmurs [15]. In fact, the differences in the accuracy rates among these four heart murmurs were statistically significant in both the first and second tests in 2010-2012 although this was not stated in our previously published article. We employed color Doppler video clips of AR and MR but not of MS because we believed that regurgitant jets of AR and MR should be much easier for the students to recognize and to associate with corresponding murmurs than video images of MS. Unexpectedly, the accuracy rates of AR and MR murmurs did not improve in 2014-2015 as compared with 2010-2012 despite the fact that we had the students refer to color Doppler video clips of AR and MR in 2014-2015 and that more than 85% of the students answered that referring to color Doppler video clips was useful or somewhat useful regarding both AR and MR.

There are several possible explanations for why referring to color Doppler video clips in the way employed in the present study did not raise the accuracy rates of AR and MR murmurs. First, color Doppler video clips were not synchronized with their corresponding heart murmurs provided by a cardiology patient simulator. Therefore, the students were not able to listen to AR and MR murmurs while watching the corresponding video clips. Students had to watch the video clips just before and after listening to AR and MR murmurs, which may have diminished the possible beneficial effects of referring to the video clips. Second, we did not evaluate how much time each student spent watching the video images. It is possible that a significant number of students might not have spared sufficient time to obtain benefits from referring to the videos. Third, we did not change the schedule of our auscultation class although color Doppler echocardiographic video clips were introduced in 2014-2015. If we had extended the time for this class, students may have had more time to spend on referring to the video images.

### Students’ favorable questionnaire responses to color Doppler echocardiographic video clips

More than 85% of students answered that referring to color Doppler echocardiographic video clips in our cardiac auscultation class was useful or somewhat useful regarding both AR and MR. This contrasts with the results of auscultation tests which showed that referring to echocardiographic video clips in the way employed in the present study did not bring any favorable effects on proficiency in accurately identifying AR and MR murmurs. Kirkpatrick and Kirkpatrick [17] proposed four levels of training evaluation, and their wording has been revised [18]. Levels 1, 2, 3, and 4 represent “the degree to which participants find the training favorable, engaging and relevant to their jobs (Reaction)”, “the degree to which participants acquire the intended knowledge, skills, attitude, confidence and commitment based on their participation in the training (Learning)”, “the degree to which participants apply what they learned during training when they are back on the job (Behavior)” and “the degree to which targeted outcomes occur as a result of the training and the support and accountability package (Results)”, respectively. Our questionnaire regarding the usefulness of referring to color Doppler echocardiographic video clips in class and the auscultation tests apply to Level 1 and Level 2, respectively. The discrepancy between the results of two different levels of training evaluation suggest that a cardiac auscultation training program favorable for participants does not necessarily provide them with proficiency to achieve goals.

Quinn et al. developed a hypothesis-driven cardiac auscultation laboratory session using a high-fidelity simulator for second-year medical students [19]. They reported that students’ evaluations of whether the session was effective had improved as faculties had become more familiar with the operation of the simulator. However, they admitted that their evaluations were Level 1 of Kirkpatrick’s learning pyramid. We believe that using only questionnaires asking about the degree of participants’ satisfaction is not sufficient to evaluate a training program and that objective evaluations of students’ proficiency in correctly identifying heart sounds and murmurs are essential to determine whether the program of cardiac auscultation is substantially useful.

### Possible unfavorable effects of referring to color Doppler echocardiographic video clips in the way employed in the present study on the auscultation test results

Overall accuracy rate of all heart sounds and heart murmurs in the first test was significantly lower and that in the second test tended to be lower in 2014-2015 than in 2010-2012. The accuracy rate of S2/S3/S4 was significantly lower in 2014-2015 than in 2010-2012 in both the first test and the second test. The accuracy rate of respiratory split S2 in the first test was significantly lower in 2014-2015 than in 2010-2012. These results suggest that referring to color Doppler echocardiographic video clips of AR and MR in the way employed in 2014-2015 not only provided no beneficial effects for proficiency in accurately identifying AR and MR murmurs but also possibly negatively affected learning of other sounds. There are several possible explanations for the unexpected results. As explained above, we did not change the schedule of our cardiac auscultation training class when we introduced color Doppler echocardiographic video clips in 2014-2015. It is possible, therefore, that students may not have been able to spend enough time listening to heart sounds and murmurs in addition to referring to the video clips. It is also possible that students may have been so distracted by the color Doppler echocardiographic videos that they may not have been able to sufficiently concentrate on listening to sounds.

### Limitations of the study

There are possible limitations in our present study that we should mention. First, as described earlier, color Doppler echocardiographic video clips used in the present study were not synchronized with their corresponding heart murmurs provided by a cardiology patient simulator. It would be helpful if we could develop color Doppler echocardiographic video clips synchronized with corresponding heart murmurs. Second, since we compared accuracy rates of all heart sounds and murmurs including those of AR and MR between 2010-2012 and 2014-2015, which were totally different groups, we have to interpret the results very cautiously. Although the schedule and learning contents of our cardiac auscultation training class in 2010-2012 and 2014-2015 were exactly the same except for referring to color Doppler video clips, the results might have differed if we had evaluated the effects of referring to color Doppler echocardiographic video clips only in students of 2014-2015 by having a control group without exposure to the video images. We did not have such a control group because we thought it could be unethical not to allow some students to be exposed to possibly beneficial learning materials. Finally, we are presenting our data several years after we finished collecting all data from our medical students. Since the results were negative in terms of developing an effective auscultation training program, it took a while for the authors to interpret the significance of our study. We do not believe, however, that our study is outdated since developing an effective cardiac auscultation training program is still an important current topic in the field of medical education [20].

## Conclusions

Referring to color Doppler video clips in the way employed in our cardiac auscultation class using a cardiology patient simulator, which most students regarded as useful, did not improve their proficiency in identifying two important regurgitant murmurs: AR and MR murmurs. More sophisticated educational materials such as video clips synchronized with their corresponding heart murmurs may contribute toward improving students’ proficiency. The results also revealed a discrepancy between students’ satisfaction and learning in our class and suggest that objective evaluations of students’ proficiency, not only questionnaires asking about the degree of their satisfaction, are essential for a cardiac auscultation training program.

## Data Availability

The datasets generated and analyzed during the current study are not publicly available due to existing ethical agreements with the participants in the present study. Data are however available from the corresponding author upon reasonable request and permission from the ethical committee.

## Abbreviations

AR: Aortic regurgitation
AS: Aortic stenosis
MR: Mitral regurgitation
MS: Mitral stenosis
S2: Second sound
S3: Third sound
S4: Fourth sound
